# Off-Label Use of Monoclonal Antibodies for Eosinophilic Esophagitis in Humans: A Scoping Review

**DOI:** 10.3390/biomedicines12112576

**Published:** 2024-11-11

**Authors:** Benyu Yang, Wenhan Li, Yiqiang Gao, Bo Zhang, Wei Zuo

**Affiliations:** 1 Department of Pharmacy, Peking Union Medical College Hospital, 1 Shuaifuyuan Wangfujing, Beijing 100730, China; benyuya@umich.edu (B.Y.);; 2 College of Pharmacy, University of Michigan-Ann Arbor, 428 Church St., Ann Arbor, MI 48109, USA; 3 State Key Laboratory of Complex Severe and Rare Diseases, Peking Union Medical College Hospital, Chinese Academe of Medical Science, Beijing 100730, China

**Keywords:** monoclonal antibody, mepolizumab, eosinophilic esophagitis, off-label, safety, FAERS, OpenVigil, systematic review

## Abstract

**Background**: Eosinophilic esophagitis (EoE) is a rare, chronic immune-mediated disorder with limited treatment options. Despite the U.S. Food and Drug Administration (FDA) approval of dupilumab for EoE, other monoclonal antibodies remain unapproved and are used off-label with limited evidence on their efficacy and safety. This systematic review rigorously and comprehensively evaluates the evidence for monoclonal antibody therapies used off-label to treat EoE. **Methods**: We conducted a systematic review across PubMed, EMBASE, Cochrane Central, and ClinicalTrials.gov, assessing the efficacy and safety of off-label monoclonal antibodies in EoE through clinical outcomes and the FDA Adverse Event Reporting System (FAERS) data. **Results**: Among ten monoclonal antibodies reviewed, mepolizumab that targets IL-5 showed the most promise with a moderate recommendation based on Level 2 evidence. Others like omalizumab (anti-IgE), dectrekumab (anti-IL-13), and reslizumab (anti-IL-5) showed limited utility. Safety evaluations via the FAERS database revealed significant adverse drug reactions, including serious events like asthmatic crises, pneumonia, and adrenal insufficiency for mepolizumab and reslizumab, as well as chronic obstructive pulmonary disease and gastroenteritis for omalizumab. Dectrekumab’s safety profile remains unclear due to a lack of data. **Conclusions**: While mepolizumab demonstrates potential as an off-label treatment, none of the antibodies reviewed have FDA approval for EoE. Clinicians should consider the balance between local and systemic effects and exercise caution, closely monitoring for adverse effects, particularly in patients with respiratory comorbidities. Continued research is crucial to establish a more robust evidence base for these therapies.

## 1. Introduction

**Figure 1:**
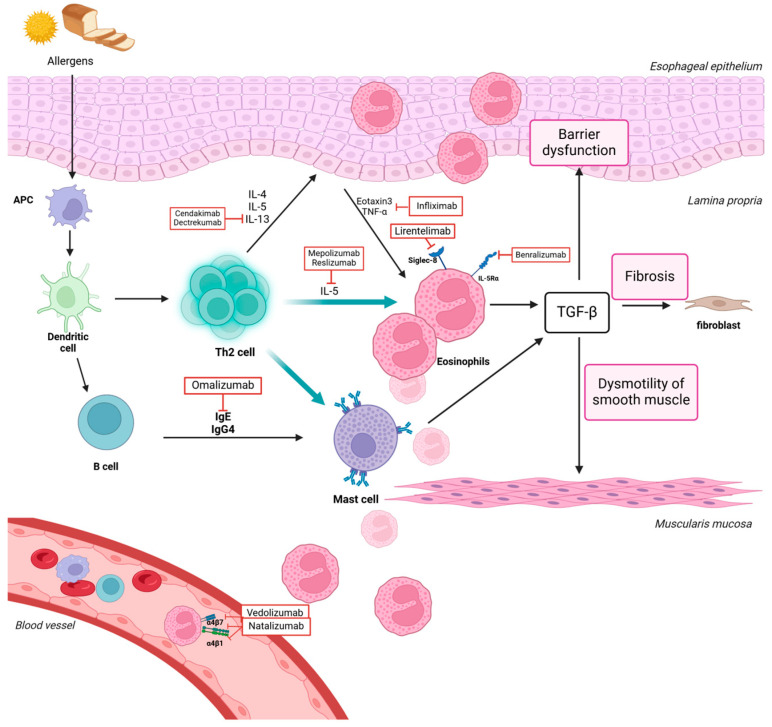
Schematic representation of the immune-mediated pathogenesis of EoE. Food or aeroallergens trigger immune activation, leading to cytokine release (IL-4, IL-5, IL-13) by Th2 cells. These cytokines promote eosinophil and mast cell activation, resulting in tissue damage, barrier dysfunction, and fibrosis in the esophagus. Monoclonal antibodies target these pathways to reduce eosinophil-mediated inflammation, with specific targets indicated.

Eosinophilic esophagitis is an immune-driven esophageal disorder that causes significant swallowing difficulties, especially in younger male patients [1]. Its incidence has been increasing in colder, rural regions [2,3]. In North America and Europe, an incidence rate of 3.7 per 100,000 patient years is estimated, with an increasing prevalence over time [4]. It is diagnosed through a combination of clinical presentation, primarily symptoms of esophageal dysfunction such as dysphagia, and the histological confirmation of tissue eosinophilia from esophageal biopsies [5]. Emerging research indicates that, alongside Th2 cytokines like IL-4, IL-5, and IL-13, immunoglobulin IgG4 might play a significant role in EoE’s pathogenesis by inducing eotaxin-3, which promotes eosinophil infiltration and activation within the esophagus (Figure 1) [6,7].

While budesonide orodispersible tablets and the recently FDA-approved dupilumab have shown efficacy, the potential for other monoclonal antibodies to treat EoE remains a critical area of research [8,9]. Before the approval of targeted therapies, commonly used empirical treatments—including swallowed topical corticosteroids, small molecule drugs like proton pump inhibitors which alter esophageal acidity and may indirectly reduce eosinophilic inflammation, biologics, and dietary restrictions—were recognized as the primary off-label therapeutic approaches for managing EoE [10,11].

This review evaluates the off-label use of monoclonal antibodies in treating EoE, focusing on agents that inhibit key pathways involved in eosinophil-mediated inflammation. For clarity, we include the specific target of each antibody in parentheses following its name. These include mepolizumab and reslizumab (IL-5), omalizumab (IgE), lirentelimab (Siglec-8), infliximab (TNF-α), dectrekumab and cendakimab (IL-13), benralizumab (IL-5 receptor α chain), vedolizumab (integrin α4β7), and natalizumab (integrins α4β1 and α4β7). We included only human clinical trials and case reports to provide a comprehensive reference for clinicians managing EoE.

## 2. Materials and Methods

### 2.1. Search Strategy

We comprehensively searched PubMed, Embase, the Cochrane Central Register of Controlled Trials (CENTRAL), and ClinicalTrials.gov to identify relevant studies up to 9 September 2024. The search strategy used the following keywords and Medical Subject Headings (MeSH) terms (“biological therapy” OR “monoclonal antibodies” OR “mepolizumab” OR “omalizumab” OR “dectrekumab” OR “QAX576” OR “benralizumab” OR “lirentelimab” OR “AK002” OR “cendakimab” OR “RPC4046” OR “reslizumab” OR “infliximab” OR “vedolizumab” OR “natalizumab” “anti-interleukin” OR “Anti-IL” OR “AntiIL”) AND (“Eosinophilic esophagitis” OR “eosinophilic oesophagitis” OR “EoE”).

### 2.2. Selection Criteria

Publications meeting the following criteria were included: (1) in English; (2) case report and case series of EoE patients; and (3) original studies, cohort studies, and registered clinical trials conducted on human subjects.

Publications were excluded if: (1) not one of the listed article types; (2) repeated clinical trial registration; (3) not related to “eosinophilic esophagitis” or “monoclonal antibody”; or (4) unavailable full-text or key information. Independent screening by two reviewers (W.L. and Y.G.) ensured objective selection, with disputes resolved through consensus.

Clinical trials were included if: (1) completed and not terminated; (2) have outcomes posted on the database.

### 2.3. Data Extraction

From the included studies, we extracted crucial details such as trial registry numbers, study types, treatments, cohorts, and primary outcomes. For case reports, patient demographics, treatment specifics, and clinical outcomes were recorded.

### 2.4. Evidence Evaluation

According to guidelines for the off-label use of medicine in China, we assessed the evidence supporting each antibody’s off-label use. This evaluation followed established frameworks like GRADE, OCEBM, and Thomson, categorizing findings into four levels of evidence—from strong recommendations for Level 1 to no recommendation for Level 4 [12].

### 2.5. Analysis of FAERS Database

We analyzed adverse event reports using OpenVigil 2 (OpenVigil 2.1-MedDRA-v24), which interfaces with the FAERS database to extract safety profiles from 2004Q1 to 2021Q3. Adverse events were categorized by MedDRA terms, and associations were quantified using the proportional reporting ratio (PRR), with established thresholds for identifying significant drug–event associations. The higher the PRR, the stronger the association. A positive signal was defined as: (1) PRR ≥ 2, χ^2^ ≥ 4 and ≥3 cases; (2) a reported odds ratio (ROR) >1; and (3) a 95% confidence interval (CI) with a ROR > 1 [13,14,15].

PRR is a statistical measure used to detect signals in pharmacovigilance, indicating the strength of association between a drug and a specific adverse event. A higher PRR suggests a stronger association. In this study, a positive signal was identified if the PRR was ≥2, indicating that the event was at least twice as likely to be reported for the drug compared to other drugs in the FAERS database.

## 3. Results

### 3.1. Systematic Review and Evidence Evaluation

**Figure 2:**
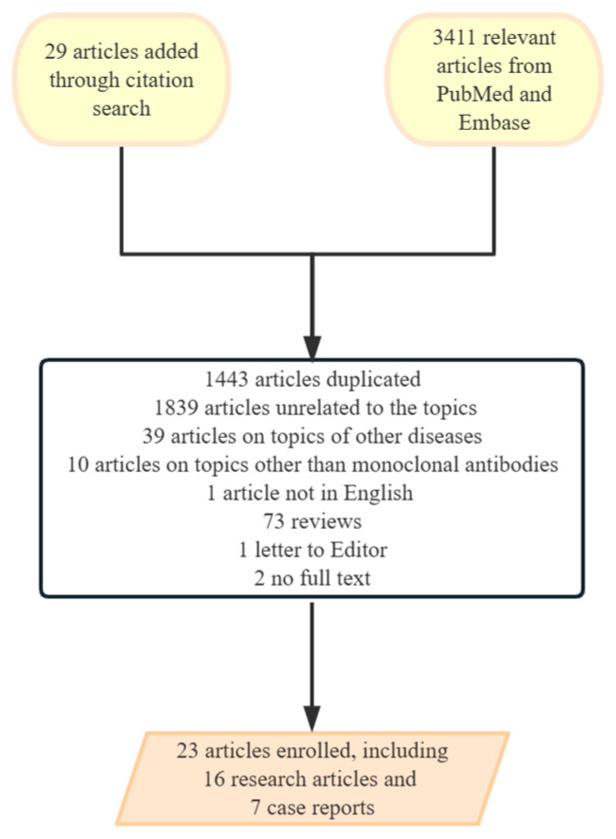
The extraction and selection process of studies and published articles. The different colors indicate stages in the selection process: yellow represents the sources of initial articles, white represents the screening and exclusion process, and orange represents the final set of articles included in the review.

**Figure 3:**
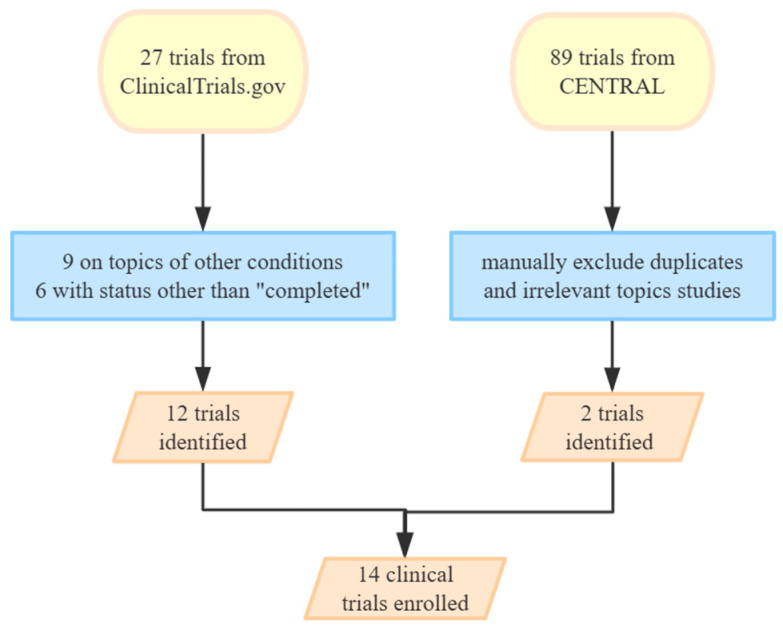
The screening and selection process of clinical trials. The different colors in the flowchart indicate distinct steps in the selection process: yellow represents initial sources, blue represents exclusion criteria or screening steps, and orange represents final included studies or trials.

Our comprehensive search of PubMed and EMBASE yielded 3408 publications, which were meticulously screened using specific keywords. Additionally, searches in CENTRAL and ClinicalTrials.gov identified 89 and 27 trials, respectively. From these sources, we selected 23 articles for detailed review. This selection included 16 publications of clinical studies and 7 case reports. Among them, 14 clinical trials were used in our final analysis, shown in Figure 2 and Figure 3.

Detailed data from clinical studies and case reports are given in Table 1 and Table 2, respectively, for reference. Table 1 provides a detailed summary of clinical study results, including study design, treatment regimens, and key outcomes for each monoclonal antibody. Table 2 summarizes individual case reports, with each row representing a single patient case, offering insights into real-world applications of the treatments.

The summary of evidence assessment is shown in Table 3. Table 3 presents the evidence assessment, with n representing the number of subjects in each group and N the total number in the study. This table highlights the strength and quality of the evidence supporting the off-label use of monoclonal antibodies in EoE.

### 3.2. Mepolizumab

Mepolizumab, which is widely approved for conditions associated with eosinophilia such as severe eosinophilic asthma and hypereosinophilic syndrome, demonstrates significant promise in the treatment of EoE [28,29,30,31]. Initial studies have shown that mepolizumab effectively reduces eosinophil counts by inhibiting the IL-5 receptor α chain on eosinophil cells [8].

Clinical Studies: Notably, a foundational open-label study conducted in 2006 (NCT00266565) on adults with EoE reported both histologic and symptomatic improvements in all four participants. Subsequent trials, including a randomized controlled trial (NCT00274703), further substantiated these findings by demonstrating reductions in TGF-β and esophageal eosinophilia among participants, although with only minor symptomatic improvements [32,33].

A study aimed at the pediatric population (NCT00358449) administered three different doses of mepolizumab, observing histological responses similar to those seen in earlier adult trials but with limited clinical symptom relief [34]. The recent post hoc analysis of this trial identified specific predictors of histological response to anti-IL-5 therapy in pediatric EoE patients. Higher BMI and the presence of exudate plaques on endoscopy were significantly associated with achieving a histologic response, defined as fewer than 15 eosinophils per high-power field or a reduction in peak eosinophil counts by 50% or more. However, the overall clinical improvement was limited, and the exact relationship between these predictors and response remains unclear, warranting further investigation [35].

A recent study (NCT03656380) aimed at adolescents and adults assessed the efficacy and safety of mepolizumab in a randomized, double-blind, placebo-controlled clinical trial. While the study did not find a statistically significant improvement in dysphagia symptoms as measured by the EEsAI compared to placebo, significant histological improvements were observed, with a reduction in esophageal eosinophil counts and endoscopic severity. At the end of three months, 42% of patients on mepolizumab achieved histological responses of less than 15 eosinophils per high-power field, compared to just 3% in the placebo group. These findings, though indicating improvements in eosinophil counts and endoscopic severity, suggest that mepolizumab may not have clinical utility in patients with severe, treatment-refractory EoE but warrants further research in less severe cases or as part of combination therapy [36].

Safety Profile: The safety profile of mepolizumab in clinical trials reflects its pharmacological mechanism. The most frequent adverse events in this cohort were gastrointestinal, such as vomiting and diarrhea, reported in the clinical trial on pediatric population (NCT00358449). An open-label study in 2006 (NCT00266565) did not record any severe adverse events (SAEs). In a more recent trial (NCT03656380), the most common adverse events were injection site reactions [36].

An analysis of the FAERS database (up to 30 June 2023) reported 18,687 SAEs and 1339 deaths associated with mepolizumab use. Respiratory-related adverse drug reactions (ADRs) were the most common, with asthma (n = 3224), dyspnea (n = 2811), and wheezing (n = 1847) frequently reported. Table 4 summarizes the top five adverse events with the highest PRR values, showing a strong association between mepolizumab use and these respiratory-related events. Newly identified signals from previous post-marketing analyses included productive cough, chest discomfort, chronic obstructive pulmonary disease (COPD), and various respiratory tract infections. The top 10 most frequent positive signals and top 10 most frequent positive signals with the highest PRR for mepolizumab are detailed in Table S1 and Table S2, repectively, in the supplementary materials.

The clinical trial data for mepolizumab indicates a generally favorable safety profile, but careful patient selection and monitoring are essential due to potentially severe respiratory reactions. This aligns with the FAERS database findings, where respiratory conditions dominate the safety signals, highlighting the need for ongoing vigilance when using mepolizumab for eosinophil-related disorders [37].

Recommendations: Despite these concerns, the overall data supports the utility of mepolizumab in managing EoE, particularly when traditional therapies are inadequate. Based on robust clinical evidence and a manageable safety profile, mepolizumab is moderately recommended for off-label use in treating EoE as per the *Management guideline for the off-label use of medicine in China* (2021) published by Peking Union Medical College Hospital (2021) [12].

### 3.3. Omalizumab

Omalizumab, as an anti-IgE antibody, is approved for managing moderate-to-severe asthma, chronic idiopathic urticaria, and allergic asthma [38]. It functions by binding to free serum IgE, preventing its interaction with high-affinity IgE receptors and thereby reducing allergic inflammation.

Clinical Studies: In the context of EoE, a first open-label trial involving 15 patients showed promising outcomes with clinical histologic remission observed in 33% of the participants (NCT01040598) [39]. Despite these encouraging results, other studies, including case reports and a randomized controlled trial involving 30 patients (NCT00123630), have shown contradictory outcomes, with no significant improvements in symptoms or histologic parameters after a 16-week treatment period [6].

Safety Profile: The first open-label trial (NCT01040598) involving 15 patients identified urticaria and respiratory tract congestion as common adverse effects of omalizumab. Another controlled trial (NCT00123630) reported inconsistent results but highlighted pneumonia, COPD, and bacterial gastroenteritis as notable ADRs.

From the FAERS database analysis up to 30 June 2023, 52,076 cases associated with omalizumab were reported, including 32,197 serious cases and 2021 deaths. The drug primarily affects the respiratory and dermatological systems, with the most frequent ADRs being asthma (n = 4843), dyspnea (n = 3925), urticaria (n = 3813), cough (n = 2586), and pruritus (n = 2254). Table 5 presents the top signals with the highest PRR which include serious conditions like respiratory tract congestion and pneumonia, emphasizing the need for vigilant monitoring. For omalizumab, the top 10 most frequent positive signals and top 10 positive signals with the highest PRR can be found in Table S3 and Table S4, respectively, in the supplementary materials.

Additional SAEs identified in real-world studies include COPD, hydrocele, and bacterial gastroenteritis, demonstrating the complex nature of omalizumab’s safety profile. This necessitates a comprehensive evaluation of risks when using omalizumab in clinical practice.

Omalizumab’s clinical trial data and the FAERS database analysis reveal a varied safety profile, with respiratory and dermatological reactions as the most common. Regular monitoring is essential to detecting SAEs early and ensuring patient safety, particularly in patients with a history of respiratory conditions [40].

Recommendations: Given the mixed evidence from clinical trials and the broad spectrum of safety concerns, the use of omalizumab for EoE should be approached with caution. Although some patients may benefit from its use, particularly where conventional therapies have failed, the decision to prescribe omalizumab should be based on a careful consideration of potential utility against the known risks. It is weakly recommended for off-label use when empirical therapy proves ineffective, although one study conducted could be considered high quality in the GRADE system. Overall omalizumab has Level 3 evidence. Additional information can be found in the Supplementary Materials. This extended dataset provides a comprehensive view of the potential adverse effects, helping clinicians make informed decisions tailored to individual patient needs [12].

### 3.4. Dectrekumab (QAX576)

Dectrekumab is a monoclonal antibody that directly targets IL-13, a cytokine secreted by Th2 cells that plays a significant role in the pathogenesis of EoE. By blocking IL-13, dectrekumab aims to reduce the inflammatory response in patients with EoE. While it has not yet been approved by any regulatory authorities for a specific indication, dectrekumab received Orphan Drug Designation from the FDA in 2013 due to its potential to treat this rare condition [8].

Clinical Studies: Awarded Orphan Drug Designation by the FDA in 2013 for its potential in EoE treatment, dectrekumab demonstrated its efficacy through a notable double-blind, placebo-controlled study conducted in 2015 (NCT01022970) [41]. In this study, 23 adult patients experienced significant symptomatic improvements, particularly in dysphagia. Additionally, there was a substantial decrease in esophageal eosinophil counts, with the treatment group showing a 60% reduction compared to a 23% increase in the placebo group (*p* = 0.004) [42]. This highlights the drug’s potential utility [12].

Safety Profile: In the 2015 double-blind study (NCT01022970), dectrekumab showed a generally favorable safety profile. The most frequent adverse events were mild, including headaches and respiratory tract infections, with no SAE documented.

Despite promising clinical results, there are no data available in the FAERS database on dectrekumab. This lack of real-world safety data underscores the necessity for further comprehensive studies to establish its long-term safety and overall tolerability. Such studies are crucial for understanding the balance between therapeutic benefits and potential risks. Future evaluations should seek to fill this knowledge gap and provide clinicians with a clearer understanding of its safety profile.

While the clinical trial data indicate that dectrekumab is generally well tolerated, more extensive research is needed to verify this in broader patient populations. Ensuring a clear understanding of dectrekumab’s long-term safety will support its careful integration into therapeutic regimens, especially for patients who have not responded to other treatments.

Recommendations: Given its demonstrated efficacy in small number of patients, and the current lack of comprehensive safety data, dectrekumab is weakly recommended for off-label use in EoE, pending further investigation.

### 3.5. Reslizumab

Reslizumab is approved only by the FDA [43] and the European Medicine Agency (EMA) [44] for add-on maintenance treatment of severe asthma with an eosinophilic phenotype [45]. Reslizumab inhibits eosinophil proliferation by targeting the IL-5 receptor’s α chain on eosinophils. In 2007, it received Orphan Drug Designation from the FDA [46].

Clinical Studies: Clinical trials have demonstrated its efficacy, with a phase III open-label study (NCT00635089) showing positive treatment outcomes [23]. Another multicenter phase II/III randomized clinical trial (NCT00538434) demonstrated a reduction in the median peak esophageal eosinophil counts by 59%, 67%, 64%, and 24%, respectively, in the 1, 2, or 3 mg/kg of reslizumab treatment and placebo groups (all *p* < 0.001). However, only limited histologic remission was shown for 226 patients in all four groups [47]. Although six SAEs occurred, the majority were in the placebo group. An open-label extension study involving 12 patients receiving reslizumab for up to 9 years showed a significant reduction in eosinophil count and symptomatic improvement [24,48].

Safety Profile: In the phase III open-label study (NCT00635089), six SAEs were reported, primarily in the placebo group. Another multicenter phase II/III trial (NCT00538434) identified lung disorders, myocardial infarction, and respiratory infections as SAEs, underscoring the need for vigilant patient monitoring. Other adverse events included mild to moderate gastrointestinal symptoms and muscle spasms.

As of 30 June 2023, the FAERS database recorded 491 cases involving reslizumab, including 277 SAEs and 13 deaths. In total, 43 ADR signals were identified. The top five signals with the highest PRR included eosinopenia and adrenal suppression (Table 6). Other frequently reported signals included drug ineffective (n = 33), asthma (n = 27), dyspnea (n = 20), wheezing (n = 11), and malaise (n = 10). The top 10 most frequent positive signals and top 10 positive signals with the highest PRR for reslizumab are presented in Table S5 and Table S6, respectively, in the supplementary materials.

Although fewer adverse events were reported for reslizumab compared to other monoclonal antibodies like mepolizumab and omalizumab, noteworthy SAEs, such as lung disorders, myocardial infarction, pneumonia, and respiratory tract infections, warrant cautious monitoring during treatment.

Reslizumab’s safety profile from clinical trials and the FAERS database highlights potential cardiovascular and respiratory risks, emphasizing the importance of careful patient selection and regular monitoring. Further research is necessary to thoroughly understand its long-term safety and guide its appropriate use.

Recommendations: Although the trials showed consistent histological responses (decreasing eosinophils in esophageal tissue), the lack of clinical symptom improvement and the small trial sizes which limited the understanding about its safety suggests a weak recommendation as an off-label treatment for EoE (Level 3 evidence) [12].

### 3.6. Lirentelimab (AK002)

Lirentelimab, a monoclonal antibody targeting sialic acid–binding immunoglobulin-like lectin 8 (CD33 receptor) on eosinophils, aims to reduce apoptosis and antibody-dependent cellular cytotoxicity [49]. Despite lacking specific approval, it received Orphan Drug Designation from the FDA in 2019 [50].

Clinical Studies: A phase II/III double-blind, placebo-controlled study (NCT04322708) in adults and adolescents with EoE showed that lirentelimab was superior to placebo in controlling inflammation. However, it did not meet one of the primary endpoints, the change in the Dysphagia Symptom Questionnaire (DSQ) score. While the trial is considered high-quality evidence, the treatment outcome was deemed ineffective. Therefore, lirentelimab is weakly recommended for off-label use in EoE.

Safety Profile: In the phase II/III clinical trial (NCT04322708), lirentelimab was generally well-tolerated, with most adverse reactions being mild to moderate. The most common adverse events included gastrointestinal discomfort and mild respiratory infections. Although the trial did not demonstrate a significant improvement in DSQ scores, lirentelimab effectively reduced inflammation, providing evidence of its potential therapeutic benefits.

While lirentelimab was shown to have a favorable safety profile, its limited efficacy in symptom relief warrants further investigation. Clinicians should consider its anti-inflammatory potential and mild side effect profile for EoE.

Recommendations: Given its demonstrated efficacy in controlling inflammation in a clinical trial, lirentelimab could be weakly recommended for off-label use in EoE (Level 3 evidence). The lack of efficacy in improving DSQ scores raises questions about its overall clinical utility. Therefore, its use should be carefully considered, weighing the potential benefits against the observed limitations in efficacy.

### 3.7. Infliximab

Infliximab is approved by the FDA, EMA, the Pharmaceuticals and Medical Devices Agency in Japan (PMDA), and the National Medical Products Administration (NMPA) in China for treating various inflammatory conditions, including Crohn’s disease, ulcerative colitis, rheumatoid arthritis, and chronic plaque psoriasis. The drug binds to TNF-α with high affinity, preventing its interaction with receptors and reducing the production of pro-inflammatory cytokines like IL-1 and IL-6 [51,52,53,54,55].

Clinical Studies: A short pilot study (NCT00523354) evaluated the off-label use of infliximab in three male adult patients with EoE that was refractory to standard treatments like proton pump inhibitors and corticosteroids. The primary outcome measured was tissue eosinophilia. Despite varying degrees of remission observed across the patients after a 4-week infliximab infusion, endoscopic evaluation did not show significant improvement [56].

Safety Profile: The small pilot study (NCT00523354) involving infliximab did not report any SAEs. However, subsequent real-world cases have documented a spectrum of reactions, including infusion reactions, opportunistic infections, and autoimmune responses, which are consistent with its known immunomodulatory effects.

Infliximab’s safety signals emphasize the need for careful patient monitoring. Although instances of tissue eosinophilia were reported in the FAERS database post-treatment, these contrasted with the lack of significant endoscopic improvement in some cases. The comprehensive analysis of FAERS data did not reveal notable safety signals specific to EoE treatment, but broader adverse reactions tied to its approved indications remain relevant, such as increased susceptibility to infections.

While infliximab’s clinical trials did not highlight significant adverse effects for EoE, real-world use indicates a range of potential risks. The delicate balance between therapeutic benefits and potential adverse effects requires clinicians to weigh the evidence carefully. The regular monitoring of patients, particularly for immunosuppressive-related effects, is crucial to ensuring the safety of infliximab treatment.

Recommendations: Given the limited efficacy demonstrated in this study and the absence of further high-quality trials, infliximab is weakly recommended for EoE treatment. It is crucial to consider the limitations of the available evidence when using infliximab as an off-label treatment for EoE.

### 3.8. Cendakimab (RPC4046)

Cendakimab (RPC4046) is a monoclonal antibody that inhibits IL-13 binding to its receptors, reducing inflammation. As RPC4046 is more prone to block the asthma phenotype of receptor, it appears more significant as a potential treatment for EoE [57]. The FDA granted it Orphan Drug Designation in 2015 due to its potential for treating EoE [58].

Clinical Studies: A randomized clinical trial (NCT02098473) involving 99 patients assessed cendakimab’s efficacy in subcutaneous doses of 180 mg or 360 mg. The results showed no significant improvement in symptoms, although there was a reduction in eosinophil counts. Common ADRs included headaches and upper respiratory tract infections [59].

Safety Profile: In the randomized clinical trial (NCT02098473), the most common adverse reactions to cendakimab were headaches and upper respiratory tract infections, indicating a mixed safety profile. No significant adverse signals were found in the FAERS database, suggesting that cendakimab is generally well-tolerated. However, the long-term safety of the drug remains to be thoroughly assessed due to limited available data. Although cendakimab shows promise as a potential treatment with a relatively mild adverse event profile, its long-term safety requires further investigation through larger, controlled trials. Clinicians should remain cautious when considering its off-label use until more comprehensive safety data are available.

Recommendations: Given the lack of promising results from clinical trials, the off-label use of cendakimab is weakly recommended and only advisable in emergency situations.

### 3.9. Benralizumab

Benralizumab is a monoclonal antibody approved by the FDA, EMA, and PMDA for severe asthma with an eosinophilic phenotype [60,61,62]. The FDA granted it Orphan Drug Designation for treating EoE. However, some cases reveal potential side effects, like urticarial rash and angioedema [63,64]. It targets the IL-5 receptor α chain on eosinophils, leading to their reduction via the FcγRIIIa receptor on natural killer (NK) cells [65,66].

Clinical Cases: In 2018, a case involving a 64-year-old female patient treated with benralizumab was reported. The patient had severe eosinophilic asthma and EoE, which showed significant improvements after benralizumab therapy. Approximately two weeks into treatment, her esophageal symptoms, such as dysphagia, disappeared, and no eosinophils were found in repeated esophageal biopsies [67]. Another 56-year-old male patient with EoE was also reported. He had been treated with topical steroids and mepolizumab before starting benralizumab therapy. As a result, there were some histologic responses, including a depletion of eosinophils in biopsy but only minor symptomatic improvements. The patient continued to require inhaled corticosteroids to manage his asthma. In this case, benralizumab reduced exacerbations and steroid dependence, but lung function did not improve [68]. Moreover, a case series of eight patients with EoE demonstrated the potential utility of benralizumab, with five of the eight patients showing endoscopic improvements and four patients achieving complete eradication of eosinophils on biopsy. [69].

More recently, a new case of a 73-year-old woman with a long history of bronchial asthma and EoE was reported. After failing to respond to prednisolone, rabeprazole sodium, and budesonide oral suspension, benralizumab was introduced in combination with these medications. Two weeks after starting benralizumab, her dysphagia completely disappeared. After six weeks, endoscopy confirmed the disappearance of multiple esophageal rings, while biopsy specimens showed no eosinophilic infiltration. The patient remained in remission even 12 months after discontinuation. This demonstrates the potential efficacy of benralizumab, particularly when combined with other medications [70].

Safety Profile: In reported case series and clinical studies, adverse reactions to benralizumab included respiratory effects like asthma, cough, and urticaria. Further investigations identified eosinophilic otitis media as a potential risk. Previous clinical trials for asthma, including the SIROCCO, CALIMA, ZONDA, and MELTEMI trials, confirmed the safety of benralizumab across various age groups. The most commonly reported adverse reactions include nasopharyngitis (11.9%), asthma (7.4%), headache (5.0%), and bronchitis (4.3%) [71,72,73]. Serious infections, hypersensitivity reactions, and malignancies were rare, and the incidence was comparable to a placebo.

According to the FAERS database, the most common adverse reactions include asthma (n = 989), dyspnea (n = 568), and cough (n = 305). Signals with the highest PRR are eosinophilic otitis media, increased fractional exhaled nitric oxide, and chronic eosinophilic rhinosinusitis. Although the adverse effects vary, they often involve significant respiratory conditions that require clinical attention.

Benralizumab’s safety profile highlights its potential for respiratory-related adverse effects, necessitating careful patient monitoring. Given its immunomodulatory nature, clinicians should remain vigilant for emerging respiratory conditions, particularly in patients with pre-existing lung conditions. In the supplementary materials, Tables S7 and S8 showed the top 10 most frequent positive signals and top 10 positive signals with the highest PRR.

Recommendations: As benralizumab only has evidence as case reports, the quality is recognized as lowest level—Level 4 [12]. Given that the evidence is based on case reports, the quality of the data is low, classified as Level 4. Therefore, benralizumab is not recommended for off-label use in EoE.

### 3.10. Vedolizumab

Vedolizumab is a monoclonal antibody approved by the FDA, EMA, and PMDA for treating moderate to severe ulcerative colitis [74,75,76]. It blocks the integrin α4β7 to prevent its interaction with mucosal vascular address in cell adhesion molecule 1. This leads to the reduction in eosinophils and overall inflammation [8].

Clinical Cases: Two case reports describe vedolizumab’s off-label use in treating EoE. One case involved a 43-year-old male with EoE and concomitant inflammatory bowel disease who experienced significant improvement in dysphagia after vedolizumab therapy. In another case, a 42-year-old female achieved complete histologic remission after six months of treatment, with zero eosinophils detected in endoscopic biopsies [77,78].

Safety Profile: In two case reports, vedolizumab was associated with adverse reactions including respiratory and gastrointestinal symptoms, such as abdominal pain and colitis.

According to the FAERS database, vedolizumab’s most frequently reported ADRs include ulcerative colitis (n = 2553), Crohn’s disease (n = 1861), and diarrhea (n = 1150). Other notable signals are abdominal pain, hematochezia, and rash. Signals with the highest PRR include a decreased therapeutic reaction time, medical device site fistula, and high-grade b-cell lymphoma.

Vedolizumab’s safety profile shows a higher prevalence of gastrointestinal adverse effects, with ulcerative colitis and Crohn’s disease as the most commonly reported reactions. Monitoring is warranted due to the potential for severe ADRs like high-grade b-cell lymphoma. The top 10 most frequent positive signals and top 10 positive signals with the highest PRR are detailed in Table S9 and Table S10, respectively, in the supplementary materials.

Recommendations: Although vedolizumab shows potential as a treatment for EoE in two cases, the evidence is based on isolated case reports, placing it at Level 4. Thus, vedolizumab is not currently recommended for off-label EoE treatment [12].

### 3.11. Natalizumab

Natalizumab is a monoclonal antibody that inhibits the integrin α4β1 and α4β7 heterodimer, reducing eosinophil recruitment to the gastrointestinal mucosa. It is approved by the EMA for relapsing-remitting multiple sclerosis and can cause adverse effects like headache, fatigue, joint, and muscle pain [79].

Clinical Cases: A case report described the use of natalizumab in a 30-year-old female patient with refractory EoE. The patient, resistant to omeprazole, fluticasone, and budesonide, also had progressive multiple sclerosis. After natalizumab treatment, she experienced symptom relief and significant histological improvements upon repeated endoscopic examinations. This outcome was attributed to the drug’s mechanism, which is similar to vedolizumab [80].

Safety Profile: In this case report, the patient treated with natalizumab developed fatigue and infusion site reactions. Controlled trials for other indications have also highlighted the risks of JC virus infection and immune suppression as notable concerns.

In the FAERS database, the most frequently reported adverse reactions include fatigue (n = 13,947), gait disturbance (n = 6230), and asthenia (n = 5422). Specific safety signals with high PRR involve the JC virus test (positive and negative), herpes zoster necrotizing retinopathy, and infusion site hematoma. Other adverse signals include poor venous access and drug delivery device implantation or removal.

The overall safety profile emphasizes the need for close monitoring due to risks of immune suppression and JC virus-related conditions. Clinicians should exercise caution, especially in patients with a history of immune disorders. The top 10 most frequent positive signals and top 10 positive signals with the highest PRR for natalizumab are presented in Table S11 and Table S12, respectively, in the supplementary materials.

Recommendations: As the evidence for natalizumab’s utility in EoE comes from a single case report, it is classified as Level 4, the lowest evidence quality. Thus, natalizumab is not recommended for off-label use in EoE [12].

## 4. Discussion

**Figure 4:**
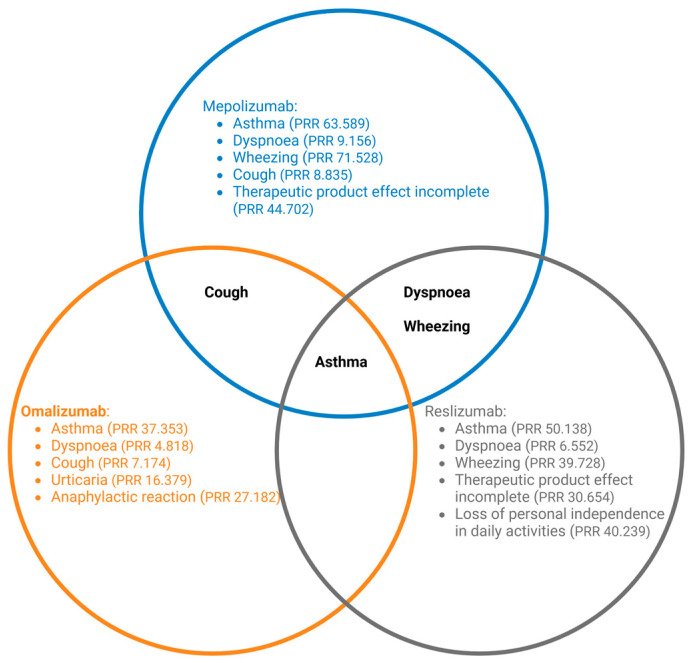
Venn diagram showing the top adverse events with the highest PRR values for mepolizumab, omalizumab, and reslizumab. Overlapping areas represent shared adverse events between the antibodies.

Mepolizumab and omalizumab have shown promising results in the treatment of EoE, with clinical trials demonstrating efficacy in reducing eosinophil counts and improving symptoms. Specifically, mepolizumab has been associated with reductions in both systemic and local eosinophil levels, which may be beneficial for patients with systemic eosinophilic involvement beyond the esophagus. In contrast, omalizumab primarily was assessed with local eosinophils within the esophagus, potentially offering a more localized effect that minimizes systemic exposure. This distinction between local and systemic action is clinically important, as it allows clinicians to tailor treatment based on the patient’s eosinophilic disease burden and comorbid conditions. However, safety concerns related to respiratory and gastrointestinal adverse effects must be considered. FAERS data revealed respiratory complications such as asthma and dyspnea, especially with mepolizumab, suggesting that while effective, these drugs require close monitoring, particularly in patients with underlying respiratory conditions (Figure 4).

Similarly, reslizumab and dectrekumab also demonstrated efficacy in reducing local eosinophil counts in clinical trials. Since their effects are mainly localized, they may pose lower systemic risks; however, post-marketing surveillance has identified hematological and respiratory events as shown in the Figure 4, highlighting the need for further investigation. The limited long-term data for these agents means that clinicians should be cautious when prescribing them off-label for EoE until more robust safety profiles are established.

Vedolizumab and natalizumab, though supported by case reports rather than large-scale trials, have shown potential in treating EoE. However, adverse events related to gastrointestinal and immune responses observed in the FAERS data suggest that their use should be approached with caution until further clinical evidence is available.

Other monoclonal antibodies, such as cendakimab, dectrekumab, and infliximab, are not discussed in detail due to the lack of sufficient clinical data or significant findings from FAERS analysis. These agents either lack substantial clinical trial results or do not show notable adverse event patterns in the FAERS database, making them less relevant for immediate clinical application in EoE.

The FAERS database provides a valuable tool for identifying adverse drug reactions, but it has several limitations. Adverse event reports come from various sources, including healthcare professionals, patients, and caregivers, leading to variability in reporting quality. Reports submitted by professionals tend to be more detailed, while those from non-professionals may omit important clinical details. Moreover, FAERS data cannot directly establish causality between drug exposure and reported events, and factors such as comorbidities, concomitant medications, and dosages can influence the reported adverse events. As a result, the data may carry biases, and further third-party analyses are often required to clarify these signals. Despite these limitations, FAERS data offer valuable insights into potential safety concerns that warrant closer monitoring [15].

## 5. Conclusions

This systematic review has evaluated the off-label use of monoclonal antibodies for EoE, highlighting both their efficacy and safety profiles. Among the antibodies studied, mepolizumab, omalizumab, reslizumab, and dectrekumab show moderate efficacy based on randomized controlled trials, making them viable options in specific clinical contexts, despite lacking formal regulatory approval for EoE. Importantly, the choice of therapy may depend on the desired balance between local and systemic effects, as this can impact both efficacy and safety profiles. However, the current evidence remains insufficient for broad clinical application without further validation, particularly for infliximab, cendakimab, benralizumab, and vedolizumab, which have shown limited but promising potential. The FAERS analysis has highlighted important safety concerns, particularly with regard to respiratory and gastrointestinal adverse events. These findings underscore the necessity for vigilant patient monitoring, especially in individuals with pre-existing respiratory conditions. While the FAERS data provide useful insights, its inherent limitations—such as reporting biases and variability—indicate a need for caution in interpreting these signals.

In clinical practice, mepolizumab, omalizumab, reslizumab, and dectrekumab may be considered as treatment options for EoE, but should be prescribed with vigilance due to associated safety risks. More comprehensive, well-controlled studies are essential to better define the role of monoclonal antibodies in EoE management. These studies will be crucial for confirming their long-term safety and efficacy, ultimately guiding clinicians in optimizing treatment for patients with EoE.

## Figures and Tables

**Table 1:** Summary of completed clinical trials for monoclonal antibodies in EoE patients. The table indicates the primary outcomes and the number of patients in each cohort (n) or the total number in the trial (N).

Name	NCT Number	Study Type	Treatment and Cohorts	Primary Outcome Measures
Mepolizumab [16]	NCT03656380	Phase 2	Mepolizumab 300/100 mg N = 66	The mean change in dysphagia score, as measured by the Eosinophilic Esophagitis Symptom Activity Index (EEsAI) decreased by 15.4 points in the mepolizumab group versus 8.3 in the placebo group (*p* = 0.14)
Mepolizumab [17]	NCT00266565	Phase 1/2	Three infusions of anti-IL-5 (750 mg intravenously monthly) N = 4	Significant blood and esophageal eosinophil reduction (20- to 30-fold; *p* < 0.0001)
Mepolizumab [18]	NCT00274703	Phase 2	Two 750 mg intravenous infusions of mepolizumab (n = 5) Placebo (n = 6)	Significant esophageal eosinophil reduction (54% at week 4, *p* = 0.03) and reduction in tenascin C and TGF-β (*p* < 0.05)
Mepolizumab [19]	NCT00358449	Phase 2	Mepolizumab (a total of three infusions): 0.55, 2.5, or 10 mg/kg every 4 weeks N = 59	Eosinophil count reduction >85%; 31% symptom improvement.
Omalizumab [20]	NCT01040598	Phase 1	Omalizumab: 2-week pre-omalizumab baseline screening. Followed by subcutaneous injection every 2 or 4 weeks for a total of 12 weeks N = 15	Significant reduction in esophageal tissue IgE levels in most patients; 33% symptom improvement.
Omalizumab [21]	NCT00123630	Phase 2	Omalizumab dosed IV based on IgE level and weight every 2–4 weeks (n = 16) Placebo (n = 14)	24–40% eosinophil reduction; no significant symptom improvement.
Dectrekumab [22]	NCT01022970	Phase 2	QAX576: 6 mg/kg (n = 17) Placebo (n = 8)	60% eosinophil reduction; improvement in dysphagia (*p* = 0.004).
Reslizumab [23]	NCT00635089	Phase 3, Open-label	Reslizumab: 1, 2, or 3 mg/kg at week 0, 4, 8 and 12 (1 mg/kg, n = 56; 2 mg/kg, n = 57; 3 mg/kg, n = 57) Placebo (n = 57)	Of 190 participants, 177 reported at least one adverse event; 7 AEs.
Reslizumab [24]	NCT00538434	Phase 2/3	Patients treated with IV reslizumab 1 (n = 55), 2 (n = 57), and 3 (n = 57) mg/kg, respectively Placebo (n = 57)	Reslizumab (1–3 mg/kg) reduced eosinophils and improved physician assessment (*p* < 0.05).
Lirentelimab [25]	NCT04322708	Phase 2/3	lirentelimab: 1 mg/kg; 6 doses given monthly lirentelimab: 1 mg/kg, followed by 5 monthly doses N = 277	Significant reduction in esophageal eosinophils (*p* < 0.0001); no significant improvement in dysphagia (*p* = 0.2470 for 1 mg/kg, *p* = 0.2372 for 3 mg/kg).
Infliximab [26]	NCT00523354	Phase 2 Proof-of-Concept Study	Infliximab: two infusions, each containing 5 mg/kg body weight at week 0 and 2. N = 3	No results posted
Cendakimab [27]	NCT02098473	Phase 2	RPC4046 (180 mg: n = 31; 360 mg, n = 34) Placebo (n = 34) Once weekly for 16 weeks	Significant eosinophil reduction in both treatment groups (*p* < 0.0001); improved dysphagia (*p* = 0.0733).

**Table 2:** Case reports of monoclonal antibody treatments in EoE patients. Each row represents an individual patient case, with improvements in histological markers and symptom resolution noted where applicable.

Drug Name	Age	Sex	Concurrent Diseases	Previous Medications	Treatment Outcome
Benralizumab	64	Female	Eosinophilic asthma	Oral steroid	Complete resolution of dysphagia and sensation of food impaction two weeks post-treatment; symptom-free with no eosinophils on biopsy months later.
Benralizumab	56	Male	Severe asthma	Oral topical steroids, mepolizumab, standard asthma treatment	After 2.5 years on mepolizumab with persistent eosinophils, benralizumab treatment led to complete histologic remission within four months.
Vedolizumab	43	Male	Crohn’s disease on the pouch, primary sclerosing cholangitis	Infliximab, ciprofloxacin	Improvement in mucosal inflammation and sustained remission of EoE symptoms (dysphagia) two months post-treatment, with concurrent pouchitis improvement and the discontinuation of ciprofloxacin.
Vedolizumab	42	Female	Ileal penetrating Crohn’s disease, asthma, allergic contact dermatitis	Infliximab, adalimumab, certolizumab	Six months of treatment led to normal esophageal mucosa and complete histologic resolution with zero eosinophils on biopsy.
Natalizumab	30	Female	Multiple sclerosis	Omeprazole, fluticasone, budesonide,	Four months post-treatment, dysphagia improved with a single proximal esophageal stricture requiring dilation. Over the following three years, the patient remained asymptomatic with normal biopsies showing no active inflammation or eosinophilic infiltration.

**Table 3:** Summary of the level of evidence and clinical recommendations for monoclonal antibody use in EoE. This table highlights the strength of evidence and clinical recommendations based on randomized controlled trials (RCTs) and case reports. Mepolizumab shows the strong evidence of RCTs and is moderately recommended. Other antibodies, like omalizumab and benralizumab, have moderate to weak evidence, highlighting the need for further research.

Drug Name	Clinical Study	^a^ Effectiveness of Evidence	^b^ Recommendation
	Phase 2 RCTs	Level 2	Moderately recommended
Mepolizumab	Phase 1/2 open-label		
Omalizumab	Phase 1 open-label	Level 2	Moderately recommended
	Phase 2 RCT		
Dectrekumab (QAX576)	Phase 2 RCT	Level 2	Moderately recommended
Reslizumab	Phase 3 Open-label	Level 2	Moderately recommended
	Phase 2/3 RCT		
Lirentelimab (AK002)	Phase 2/3 RCT	Level 3	Weakly recommended
Infliximab	Phase 2 open-label	Level 3	Weakly recommended
Cendakimab (RPC4046)	Phase 2 RCT + open label	Level 3	Weakly recommended
Benralizumab	Two case reports	Level 4	Not recommended
Vedolizumab	Two case reports	Level 4	Not recommended
Natalizumab	Case report	Level 4	Not recommended

^a^: Effectiveness of evidence is categorized by levels. Level 1: Strong evidence from RCTs with consistent results across multiple studies. Level 2: Moderate evidence from phase 1 or 2 trials, or open-label studies, with some limitations in study design or sample size. Level 3: Limited evidence from observational studies, non-randomized trials, or inconsistent results. Level 4: Weak or anecdotal evidence based on case reports or expert opinion without robust clinical trials. ^b^: Recommendations are categorized as follow: strongly recommended—based on consistent evidence from multiple high-quality RCTs; moderately recommended—supported by phase 2 or smaller clinical trials, but more robust data are needed; weakly recommended: evidence is limited or inconsistent; further studies are required before definitive conclusions can be made; not recommended—insufficient or weak evidence, not currently recommended for clinical use in EoE.

**Table 4:** The top five most frequent positive signals with the highest PRR for mepolizumab.

PT	PRR	Cases	χ^2^
*CFTR* gene mutation	6254.765	8	4884.04
broncholithiasis	4300.151	11	6626.623
Inspiratory capacity abnormal	3127.38	16	9384.97
plethysmography	2345.54	3	1219.76
fungal test	1563.69	6	2623.6

PT = preferred terms; PRR = proportional reporting ratio; χ^2^ = chi-square.

**Table 5:** The top five most frequent positive signals with the highest PRR for omalizumab.

PT	PRR	Cases	χ^2^
Human antibody test	879.75	3	456.33
Pleural rub	684.25	14	2663.48
Allergy to fermented products	586.5	8	1368.99
Sputum culture	502.714	12	2032.11
Blood pressure ambulatory abnormal	488.75	5	738.357

PT = preferred terms; PRR = proportional reporting ratio; χ^2^ = chi-square.

**Table 6:** The top five most frequent positive signals with the highest PRR for reslizumab.

PT	PRR	Cases	χ^2^
Eosinopenia	4870.21	3	9556.2
Adrenal suppression	912.234	5	3643.91
Chronic spontaneous urticaria	909.106	4	2745.85
Eosinophilic granulomatosis with polyangiitis	567.179	4	1717.83
Pulmonary granuloma	483.078	3	995.415

PT = preferred terms; PRR = proportional reporting ratio; χ^2^ = chi-square.

## Data Availability

Data are available upon request to the corresponding author.

## Supplementary Materials

The following supporting information can be downloaded at: https://www.mdpi.com/article/10.3390/biomedicines12112576/s1; Table S1: Top 10 most frequent positive signals for mepolizumab; Table S2: Top 10 positive signals with the highest PRR for mepolizumab; Table S3: Top 10 most frequent positive signals for omalizumab; Table S4: Top 10 positive signals with the highest PRR for omalizumab; Table S5: Top 10 most frequent positive signals for reslizumab; Table S6: Top 10 positive signals with the highest PRR for reslizumab; Table S7: Top 10 most frequent positive signals for benralizumab; Table S8: Top 10 positive signals with the highest PRR for benralizumab; Table S9: Top 10 most frequent positive signals for vedolizumab; Table S10: Top 10 positive signals with the highest PRR for vedolizumab; Table S11: Top 10 most frequent positive signals for natalizumab; Table S12: Top 10 positive signals with the highest PRR for natalizumab.
