# Supplementary material for: Off-Label Use of Monoclonal Antibodies for Eosinophilic Esophagitis in Humans: A Scoping Review
Source: Biomedicines. 2024 Nov 11;12(11):2576. doi: 10.3390/biomedicines12112576 (PMC11592289; doi:10.3390/biomedicines12112576)
Supplement: Supplementary file 1 [file biomedicines-12-02576-s001.zip › biomedicines-3231691-supplementary.pdf]

Table S1. The top 10 most frequent positive signals for **mepolizumab**.

LLT	Cases	PRR	χ 2
asthma	3224	63.589	184370
dyspnoea	2811	9.156	20657
Wheezing	1847	71.528	117860
cough	1210	8.835	8392.15
Therapeutic product effect incomplete	1404	44.702	56840.5
loss of personal independence in daily activities	1287	69.224	79554.1
hospitalisation	541	6.113	2391.73
product dose omission issue	561	8.867	6454.67
pneuomonia	965	6.316	4481.49
Sleep disorder	929	251.578	182093

LLT = Lowest level term, PRR = proportional reporting ratio, χ 2 = chi-square.

Table S2. The top 10 most frequent positive signals with the highest PRR for **mepolizumab**.

PT	PRR	Cases	χ 2
Cftr gene mutation	6254.765	8	4884.04
broncholithiasis	4300.151	11	6626.623
inspiratory capacity abnormal	3127.38	16	9384.97
plethysmography	2345.54	3	1219.76
fungal test	1563.69	6	2623.6
sputum culture	1340.31	12	5432.99
bronchial neoplasm benign	1172.77	3	974.809
vital capacity decreased	657.304	95	33465.3
flail chest	586.384	12	3679.29
dupuytren's contracture operation	521.23	4	953.217

PT = preferred terms, PRR = proportional reporting ratio, χ 2 = chi-square.

Table S3. The top 10 most frequent positive signals for **omalizumab**.

LLT	Cases	PRR	χ 2
asthma	4843	37.353	152577
dyspnoea	3925	4.818	11955.1
Cough	2586	7.174	13546.9
Urticaria	3813	16.379	52493.1
Pruritus	2254	4.817	6793.69
No adverse events	2744	10.316	22465.3
Fatigue	2305	2.071	1307.42
Malaise	2195	3.082	3116.23
Headache	2160	2.234	1501.09
Anaphylactic reaction	1953	27.182	45148.6

LLT = Lowest level term, PRR = proportional reporting ratio, χ 2 = chi-square.

Table S4. The top 10 most frequent positive signals with the highest PRR for **omalizumab**.

PT	PRR	Cases	χ 2
Human antibody test	879.75	3	456.33
Pleural rub	684.25	14	2663.48
Allergy to fermented products	586.5	8	1368.99
Sputum culture	502.714	12	2032.11
Blood pressure ambulatory abnormal	488.75	5	738.357
Chronic spontaneous urticaria	470.062	218	39021.8
Forced expiratory flow decreased	469.2	24	4133.85
Forced expiratory volume decreased	447.386	685	120634
Fev1/fvc ratio decreased	432.158	28	4695.75
Smoke sensitivity	404.033	62	10313.7

PT = preferred terms, PRR = proportional reporting ratio, χ 2 = chi-square.

Table S5. The top 10 most frequent positive signals for **reslizumab**.

LLT	Cases	PRR	$\chi 2$
Drug ineffective	33	3.974	33
asthma	27	50.138	27
dyspnoea	20	6.552	20
wheezing	11	39.728	11
malaise	10	3.77	10
Therapeutic product effect incomplete	10	30.654	10
Loss of personal independence in daily activities	8	40.239	8
myalgia	10	10.032	10
Headache	9	2.506	9
urticaria	8	8.83	8

LLT = Lowest level term, PRR = proportional reporting ratio, $\chi 2$ = chi-square.

Table S6. The top 10 most frequent positive signals with the highest PRR for **reslizumab**.

PT	PRR	Cases	$\chi 2$
Eosinopenia	4870.21	3	9556.2
Adrenal suppression	912.234	5	3643.91
Chronic spontaneous urticaria	909.106	4	2745.85
Eosinophilic granulomatosis with polyangiitis	567.179	4	1717.83
Pulmonary granuloma	483.078	3	995.415
Mycobacterium avium complex infection	415.717	5	1663.92
Paradoxical drug reaction	175.891	4	530.606
Upper-airway cough syndrome	108.08	4	323.682
Bronchiectasis	105.733	3	215.066
Frustration tolerance decreased	95.494	4	285.241

PT = preferred terms, PRR = proportional reporting ratio, $\chi 2$ = chi-square.

Table S7. The top 10 most frequent positive signals for **benralizumab**.

LLT	Cases	PRR	χ 2
asthma	989	42.602	39371.5
dyspnoea	568	4.229	1426.91
cough	305	5.095	1007.29
Wheezing	265	21.956	5226.97
headache	394	2.491	359.504
pyrexia	242	3.155	358.199
product dose omission issue	189	4.125	446.769
rash	226	2.24	156.34
pruritus	186	2.409	153.674
arthralgia	182	2.041	97.017

LLT = Lowest level term, PRR = proportional reporting ratio, χ 2 = chi-square.

Table S8. The top 10 most frequent positive signals with the highest PRR for **benralizumab**.

PT	PRR	Cases	$\chi 2$
Eosinophilic otitis media	1202.53	4	2205.11
Fractional exhaled nitric oxide increased	1002.11	5	2602.96
Chronic eosinophilic rhinosinusitis	676.424	3	1021.03
Fev1/fvc ratio decreased	315.664	7	1610.32
Forced expiratory volume abnormal	219.975	5	785.967
Eosinopenia	150.316	4	418.431
Eosinophil count abnormal	137.171	20	2387.54
Eosinophilic granulomatosis with polyangiitis	126.402	37	4185.51
Sars-cov-2 test negative	66.262	9	496.8
Symptom recurrence	63.21	41	2365.78

PT = preferred terms, PRR = proportional reporting ratio, $\chi 2$ = chi-square.

Table S9. The top 10 most frequent positive signals for **vedolizumab**.

LLT	Cases	PRR	χ 2
off label use	3376	8.132	21537
no adverse event	636	6.01	2650.6
therapeutic reaction time decreased	487	3544.71	302999
colitis ulcerative	2553	188.746	381937
crohn's disease	1861	81.083	133127
diarrhoea	1150	3.329	1911.34
abdominal pain	991	7.973	6027.94
haematochezia	867	34.533	27018.7
frequent bowel movements	646	65.771	37881.6
arthralgia	597	2.817	706.247

LLT = Lowest level term, PRR = proportional reporting ratio, χ 2 = chi-square.

Table S10. The top 10 most frequent positive signals with the highest PRR for **vedolizumab**.

PT	PRR	Cases	χ 2
Therapeutic reaction time decreased	3544.71	487	302999
Medical device site fistula	1009.31	4	1321.22
b-cell unclassifiable lymphoma high grade	540.701	5	1271.79
Loss of therapeutic response	425.493	172	46360.9
Cervical friability	283.868	3	426.248
Drug metabolizing enzyme increased	189.245	3	311.256
Colitis ulcerative	188.746	2553	381937
Gastrointestinal stoma output abnormal	181.675	6	729.648
Arthritis enteropathic	158.722	13	1556.29
Bile acid malabsorption	153.442	15	1764.08

PT = preferred terms, PRR = proportional reporting ratio, χ 2 = chi-square.

Table S11. The top 10 most frequent positive signals for **natalizumab**.

LLT	Cases	PRR	$\chi 2$
fatigue	13947	3.883	29513
gait disturbance	6230	7.042	30130.8
asthenia	5422	3.156	7820.9
multiple sclerosis relapse	10061	33.934	232597
headache	6992	2.199	4581.71
multiple sclerosis	5955	25.933	110408
memory impairment	5231	7.545	27510.7
fall	4845	3.229	7280.89
balance disorder	3899	9.785	27758.5
urinary tract infection	3955	5.727	14568.9

LLT = Lowest level term, PRR = proportional reporting ratio, $\chi 2$ = chi-square.

Table S12. The top 10 most frequent positive signals with the highest PRR for **natalizumab**.

PT	PRR	Cases	χ 2
Jc virus test	1665.15	19	1496.96
Herpes zoster necrotising retinopathy	262.918	3	135.068
Suprapubic catheter insertion	262.918	3	135.068
Infusion site haematoma	145.714	138	7394.04
Marburg's variant multiple sclerosis	116.852	8	345.193
Drug delivery device implantation	112.51	95	4548.15
Jc virus test positive	93.443	322	14207.8
Neurostimulator removal	87.639	3	88.398
Poor venous access	87.052	1481	63175.4
Drug delivery device removal	73.033	5	156.062

PT = preferred terms, PRR = proportional reporting ratio, χ 2 = chi-square.
